# Fibrin-Based Biomaterials in Wound Healing and Soft Tissue Regeneration: Biological Mechanisms and Clinical Applications

**DOI:** 10.3390/gels12070604

**Published:** 2026-07-07

**Authors:** Bogdan Mircea Măciuceanu Zărnescu, Elena-Theodora Moldoveanu, Adelina-Gabriela Niculescu, Alexandru Scafa Udriște, Alexandru Mihai Grumezescu, Sebastian Vâlcea

**Affiliations:** 1 University of Medicine and Pharmacy “Carol Davila”, 8, Eroii Sanitari, 050474 Bucharest, Romania; bmaciuceanu@gmail.com (B.M.M.Z.); alexandru.scafa@umfcd.ro (A.S.U.); sebastian.valcea@gmail.com (S.V.); 2 Faculty of Chemical Engineering and Biotechnologies, National University of Science and Technology Politehnica Bucharest, Gh. Polizu St. 1–7, 060042 Bucharest, Romania; elena.moldoveanu99@upb.ro (E.-T.M.); agrumezescu@upb.ro (A.M.G.); 3 Research Institute of the University of Bucharest—ICUB, University of Bucharest, 050657 Bucharest, Romania

**Keywords:** chronic wounds, wound healing, fibrinogen, fibrin, fibrin-based materials, clinical applications

## Abstract

Given the prevalence of chronic wounds and soft tissue defects, which are associated with major complications such as persistent inflammation, poor vascularization, infection risk, and delayed tissue remodeling, there is a need for new materials that can overcome these limitations. Fibrin-based materials have attracted researchers’ attention for their roles in hemostasis and wound healing, as well as their biocompatibility, biodegradability, and ability to mimic the extracellular matrix. Regarding the clinical applicability of fibrin-based materials, they are currently available on the market as fibrin sealants. However, efforts are underway to improve their properties by developing hydrogels, platelet-derived fibrin matrices, and composite scaffolds that enhance mechanical stability, bioactivity, and the controlled release of cells or therapeutic agents. In addition, the number of clinical studies and registered clinical trials reflects interest in the potential applicability of fibrin-based materials in medical applications. However, the available clinical evidence remains limited for many emerging systems, and further validation is required. Although significant limitations remain, including rapid degradation, variable mechanical strength, and the need for standardized manufacturing processes, recent advances in hybrid systems and biofabrication technologies suggest promising future potential for personalized regenerative therapies.

## 1. Introduction

Chronic wounds involve a superficial to full-thickness skin loss and are known for their failure to heal within an expected time frame, representing a worldwide clinical burden due to their increasing incidence [1]. Their prevalence increases with age, and they are considered a serious threat to the elderly population [1,2]. Complications in chronic wounds are associated with other conditions that include increased glycemia, neuropathy, and impaired vascularization. These comorbidities hinder the normal healing process, imply higher healthcare costs, and affect resource allocation and evaluation of treatment strategies [2,3]. In general, chronic wounds are associated with complications and increased morbidity and considered a socioeconomic burden.

From a clinical perspective, chronic wounds are characterized by an abnormal wound-healing process and fail to progress through the orderly, timely stages of healing. They can be classified into major categories based on their etiology: arterial, venous, diabetic, and pressure ulcers (AU, VU, DU, and PU) [4,5,6]. Despite their classification, chronic wounds share common pathological features, including persistent inflammation, infection, microbial biofilm formation, and cells unresponsive to healing stimuli [4]. A wound is considered chronic if it fails to heal within 4 to 12 weeks [6,7].

It was reported that chronic wounds affect approximately 1–2% of the population in developed countries and represent a growing global health and economic burden [6,8]. Their prevalence is observed among older adults, women, and patients with diabetes and is associated with an increased rate of complications such as diabetic foot ulcers, increasing the risk of amputation and mortality [9,10]. However, the incidence of chronic wounds continues to increase, with ulcers accounting for the majority of lower limb amputations [11,12].

Effective wound healing requires an optimal wound environment and control of wound bed moisture. These attributes further facilitate wound healing through cell communication, improved epithelial migration, enhanced collagen deposition, and enhanced autolytic debridement [13]. Traditional wound dressings, such as gauze, can serve as a primary protective barrier against the external environment, but they are unable to maintain the wound’s optimal moisture [14]. Consequently, they adhere to the wound bed, causing tissue damage and pain during the frequent removal and replacement [14,15,16]. Their limited ability to maintain an optimal healing environment increases the risk of infection and makes them less suitable for chronic wounds [8,15,16]. Furthermore, traditional dressings lack real-time adjustment to therapeutic delivery or biological factors, resulting in delayed healing outcomes [17]. Furthermore, traditional wound dressings fail to adapt to changes in wound conditions and their dynamic parameters, such as pH, temperature, and humidity, nor do they support real-time therapeutic adjustments, and fail to modulate therapeutic functions according to wound needs, which may delay the healing of complex, chronic wounds [18,19].

Due to the limitations of traditional dressings, interest in developing new, more effective strategies for wound management has spurred research into biomaterials that have evolved from inert to bioactive, promoting tissue regeneration and possessing anti-inflammatory, antimicrobial, and hemostatic properties designed to enhance wound healing [20,21]. In this sense, biomaterials are designed to mimic the extracellular matrix (ECM), thereby improving cell attachment, proliferation, and differentiation, meeting several criteria such as biocompatibility, biodegradability, appropriate mechanical properties, and non-toxicity [22,23,24]. This approach enhances tissue regeneration and enables the design of more effective therapeutic strategies for complex wounds [25]. Using key components present in ECM (e.g., collagen, fibrin) represents a crucial role in the development of such biomaterials. In this context, ECM-like components are matrices that support structural features to enhance cellular processes involved in wound healing. Fibrin, an ECM component, can serve as a temporary scaffold and is also involved in the early stages of wound healing. It can promote cell migration and interactions [22,23]. Integration of natural and synthetic polymers can be used to develop a variety of dressings to support wound healing. They enable the creation of tailored structures and functional surfaces suitable for diverse wound environments [26]. In the development of wound dressings, natural polymers have gained particular attention due to their biodegradability, biocompatibility, and similarity to native biological structures, making them more attractive than synthetic materials and leading to their use in the fabrication of bioactive materials, drug delivery systems, and scaffolds [27,28]. In addition, their intrinsic properties (e.g., antimicrobial and anti-inflammatory effects) make them suitable for wound care, especially for chronic wounds, given the challenges posed by persistent infection and inflammation [29].

Scaffolds represent a key innovation in this field and can be manufactured from natural, synthetic, and hybrid materials, serving as platforms for delivering cells, drugs, and growth factors to the wound site, promoting tissue regeneration and healing. These cellular or acellular systems can create controlled microenvironments that regulate cellular behavior and enhance tissue repair by mimicking the ECM, thereby improving healing outcomes [30,31,32]. Moreover, using a polymeric matrix that gradually degrades leaves only the new tissue, and minimizes the potential tissue damage [33].

However, an ideal biomaterial designed for wound healing should maintain moisture balance, allow gas exchange, prevent infection, and support controlled drug delivery. Moreover, materials used in scaffold development should have controlled biodegradability that matches the rate of tissue regeneration, preserves drug pharmacokinetics, and ensures controlled release at a desired rate [34].

Fibrinogen and fibrin gained interest due to their important roles in wound healing. These components are actively involved in important functions, including regulating blood clotting, fibrinolysis, cellular and matrix interactions, the inflammatory response, wound healing, and neoplasia. In addition, fibrin’s multifunctional biological properties make it a key target for the design of advanced wound-healing therapies [23,35].

Thus, this paper explores the role of fibrin-based materials in wound healing and soft tissue remodeling with particular emphasis on their biological mechanisms, material design strategies, therapeutic potential, and their prospective clinical translation. Therefore, this paper was structured as a narrative review and conducted through a comprehensive literature search of relevant research on fibrin-based materials used in wound healing and regenerative medicine. The literature search implied the use of bibliographic databases such as PubMed, Scopus, and Web of Science, as well as search tools and publisher platforms such as Google Scholar, MDPI, ScienceDirect, SpringerLink, and Wiley Online Library. To provide a solid scientific context, landmark and fundamental studies have also been included, with a particular emphasis on recent publications available through April 2026, thereby illustrating the evolution of fibrin-based biomaterials in the field of wound healing and tissue regeneration. The search strategy employed combinations of the following keywords: “fibrin”, “fibrinogen”, “fibrin-based biomaterials”, “fibrin sealants”, “fibrin hydrogels”, “fibrin scaffolds”, “wound healing”, “chronic wounds”, “soft tissue regeneration”, “drug delivery”, and “tissue engineering”. As for the publications, they were selected based on their relevance to the biological functions of fibrin, the development and characterization of fibrin-based materials, and their potential for application in fields such as wound healing and regenerative medicine, as well as for clinical translation. Original research articles addressing the design, development, and characterization of fibrin-based materials, clinical trials, and relevant records available through ClinicalTrials.gov were included in the review. Studies were excluded if they were not directly relevant to fibrin-based materials, were not relevant to the scope of fibrin-based materials, did not provide information regarding the scope of this paper, or were conference abstracts, editorials, or not available in English.

The present work aims to highlight the biological roles of fibrin in hemostasis, modulation of inflammation, cell migration, angiogenesis, and its capacity to mimic the extracellular matrix. In this regard, studies from all publication years were considered, focusing on fundamental contributions that have advanced research on fibrin in wound healing and its potential applications in tissue regeneration, as well as recent advances in fibrin-based materials, including hydrogels, fibrin-based adhesives, and scaffolds. Moreover, the review highlights the properties of fibrin, including excellent biocompatibility, biodegradability, bioactivity, and extracellular-matrix-mimicking characteristics. However, drawbacks such as rapid degradation, relatively poor mechanical strength, and limited long-term structural stability hinder their translation into clinical applications. Consequently, a major objective in developing fibrin-based materials is to preserve fibrin’s biological advantages while overcoming its intrinsic limitations through materials engineering. Thus, key insights into emerging strategies, including composite biomaterials, controlled drug delivery, and 3D bioprinting, that may enhance the next generation of fibrin-based regenerative therapies are also addressed throughout this work.

## 2. Biological Basis of Fibrin in Wound Healing

**Figure 1:**
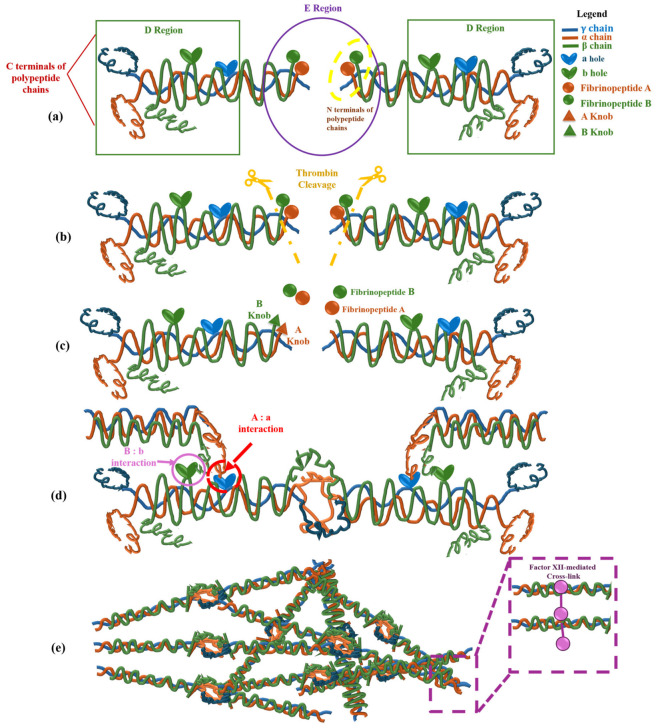
Schematic representation of the structure of fibrinogen and its conversion to fibrin. (**a**) Structure of fibrinogen Aα (orange), Bβ (green), and γ (blue); (**b**) Thrombin cleavage and release of fibrinopeptides A and B. (**c**) Depiction of the A-knob and B-knob binding sites following the removal of fibrinopeptides A and B and the formation of the active fibrin monomer. (**d**) Fibrin polymerization through specific interactions between knob A and a hole, and knob B and b hole. (**e**) Fibirn fibers formation. Created based on the information from [43,44].

Fibrinogen and fibrin represent key factors involved in the hemostasis phase, playing essential roles in blood coagulation, wound healing, inflammation, and tissue regeneration [35,36,37]. Fibrinogen (Figure 1a), a soluble plasma glycoprotein synthesized in the liver, composed of three pairs of polypeptide chains (Aα, Bβ, and γ), encoded by genes FGA, FGB, and FGG, is converted into fibrin due to the action of thrombin, which is triggered by vascular injuries [36,37,38]. The thrombin-mediated cleavage (Figure 1b) of fibrinopeptides A and B, from the N-terminal ends of Aα and Bβ chains, creates binding sites that enable the intermolecular “knob–hole” interactions (Figure 1c) [37,39,40]. Thus, these “knobs” interact with complementary “holes” presented in the globular domains of other fibrin molecules, due to A:a interactions, which represent the first step in fibrin polymerization (Figure 1d) [37,39]. In this context, resulting fibrin monomers polymerize via noncovalent interaction between D and E domains, followed by subsequent lateral aggregation, conducted by intermolecular crosslinking of α chains and probably by interactions between α and γ chains [39,41]. These interactions lead to the formation of fibrin monomers into protofibrils, generating a three-dimensional fibrin network (Figure 1e) [39,41,42]. The resulting hierarchical structure, an organized network that possesses viscoelastic and strain-stiffening properties, forms a stable blood clot that prevents severe blood loss [36,37,38,39,41,42]. The mechanical stability of the fibrin network is also improved by covalent cross-linking via factor XIII (factor XIIIa) activation, which stabilizes γ–γ and α–α chain interactions, resulting in a clot with increased stiffness and reduced susceptibility to fibrinolysis [38,39].

Fibrin’s mechanical and structural properties are strongly influenced by network architecture and biochemical conditions. In this case, fibrinogen and thrombin concentrations, ionic strength, and pH influence the fiber’s thickness, density, and porosity, as well as the clot’s overall mechanical properties [45,46]. Thus, a dense network of thin fibers, produced due to high concentration of thrombin, has greater resistance to fibrinolysis than a coarse network of thicker fibers, due to low concentration of thrombin, which are more prone to degradation [45]. Fibrin regulates this process by binding fibrinolytic components such as plasminogen and tissue-type plasminogen activator, while degradation is governed by plasmin and neutrophil elastase [47,48]. Moreover, fibrin fibers exhibit high extensibility and reversible deformation, while the network as a whole has viscoelasticity and self-repairing behavior under mechanical stress [49].

In hemostasis, fibrin, together with platelets, creates a stable hemostatic plug. Fibrin interacts with platelets, promoting their adhesion and aggregation, thereby forming a clot that not only seals the wound but also contracts under platelet-generated forces [35,50,51]. Moreover, platelets are important in this step because they are responsible for growth factor (GF) release (e.g., platelet-derived growth factor (PDGF), transforming growth factor beta (TGF-β)). These GFs are responsible for the initiation and regulation of the following phases in tissue repair [35,50,52]. Thus, the fibrin network protects GF from degradation and improves the local cellular signaling [53]. However, fibrin does not merely play a role in coagulation; it can also serve as a temporary ECM, a fibrin-rich polymer with interspersed, crosslinked plasma fibronectin, which is directly involved in the wound healing process [51,54,55,56]. In this sense, fibroblasts are involved in the development of the ECM’s new matrix, governed by cytokines (e.g., interferon-gamma (IFN-γ) and transforming growth factor-beta (TGF-β)), which contribute to collagen and fibronectin synthesis and restore tissue integrity and mechanical strength [52]. The resulting scaffold promotes the adhesion, migration, proliferation, and differentiation of cells, including neutrophils, macrophages, fibroblasts, endothelial cells, and keratinocytes [51,53,55]. In the early stages of the healing process, immune cells can penetrate the fibrin matrix and prevent the adhesion of pathogens and debris. This is followed by the recruitment and activation of cells that initiate tissue generation and promote angiogenesis [51].

In this process, fibrin is continuously remodeled, and its degradation results from cellular activity. This dynamic action ensures the transition from a temporary fibrin-based ECM to a mature tissue structure. However, dysregulation of fibrin architecture or its interactions with growth factors can impair healing and contribute to pathological conditions such as chronic inflammation or fibrosis [48,57]. Proinflammatory M1 macrophages contribute to matrix remodeling by secreting MMP9, which degrades fibrin and the ECM, thereby generating danger-associated molecular patterns (DAMPs). DAMPs can trigger and amplify the inflammatory response [57].

## 3. Fibrin-Based Biomaterials: Types, Design Strategies, and Clinical Applications

### 3.1. Fibrin Sealants

Fibrin sealants (FSs), also known as fibrin glues, are used as biological adhesives in medical practice. These materials are designed to mimic the final stages of the natural coagulation cascade and to promote hemostasis and favor tissue healing [35,58,59]. FSs are composed of fibrinogen and thrombin, which interact upon application to form an insoluble fibrin network at the injury site [58]. The FS biomimetic mechanism improves the integration of sealants into host tissue, making them highly biocompatible and absorbable, and minimizing the inflammatory response and tissue necrosis [35]. FS functionality is determined by the concentration of its components. Mechanical and structural features of the clot are influenced by the fibrinogen concentrations. Thus, it was observed that fibrinogen at increased concentrations generates the clot’s stiffness and improves adhesion. Thrombin regulates the polymerization rate, thereby affecting how quickly the sealant forms a stable network [60]. In addition, factor XIII contributes to improved mechanical stability through covalent cross-linking, while calcium ion concentration favors clot formation [58,60].

Fibrin-based sealants are used in different medical fields, including cardiovascular, orthopedic, dermatologic, and ophthalmologic surgeries, as well as in burn management and skin grafting [58,61,62,63]. In general, FSs are used to control diffuse or oozing bleeding and in procedures where conventional suturing is ineffective or impractical [61]. In general, FSs are applied via spray systems or dual-syringe devices, enabling minimally invasive use of the medical devices and reducing operation time and the need for additional mechanical fixation methods [60,61]. FSs can be found in various formulations. In dual-syringe applicators, fibrinogen and thrombin are combined at the wound site, whereas spray systems ensure even distribution. Powder-based formulations are composed of autologous FSs produced directly from the patient’s blood. Moreover, fibrin-based patches combine polymeric matrices with clotting components, improving the material’s adherence and integration into tissues [60].

Despite their hemostatic behavior, FS can serve as a bioactive platform for various therapeutic agents. GF, drugs, or cells can be introduced into the fibrin matrix, allowing a controlled release and localized therapeutic effects. Embedding such agents into fibrin systems can significantly enhance their bioavailability and efficacy for wound healing and tissue regeneration [35]. However, even if FSs offer advantages, they also have several limitations. In this regard, FSs fail to meet clinical requirements and provide insufficient mechanical support in high-stress environments, whereas products derived from pooled human plasma carry a minimal risk of disease transmission despite rigorous screening processes. Other challenges include high production costs, limited shelf life, and the potential for allergic reactions due to additives such as antifibrinolytic agents [61,64,65]. FS use can be associated with increased risk of thrombosis following intravascular administration, hypersensitivity reactions, and gas embolism during spray application (Table 1). Moreover, because FSs are usually obtained from human plasma, they carry a residual risk of pathogen transmission, including viral infections such as HIV. Another drawback of FSs is their rapid in vivo degradation via fibrinolysis, which can compromise their long-term effectiveness. However, even if antifibrinolytic agents can delay FS degradation, adverse reactions, such as allergic or anaphylactic reactions, can limit their use in sealant development [60,66].

The first generation of FSs used animal-derived components, such as bovine aprotinin, which were associated with adverse immune reactions, whereas modern formulations now use human-derived or recombinant components. The development of new strategies to improve FSs focuses on enhancing mechanical performance, adhesion strength, and functional versatility while maintaining their inherent biocompatibility [65,67].

Table 1 lists commercially available fibrin-based products used in medical practice, particularly in surgical procedures, highlighting their composition, application methods, indications, and adverse effects. Notably, most of these adhesives use fibrinogen and thrombin, essential components of the coagulation cascade, thereby mimicking the physiological mechanisms of hemostasis. It can be observed that fibrin-based adhesives are primarily used to prevent bleeding, promote tissue adhesion, and support sutures. Their main applications are in vascular, plastic, and general surgery. Overall, the table highlights the effectiveness of these products, but also the need for their prudent use.

**Table 1:** Overview of clinically available fibrin-based sealants.

Commercially Available Product	Producer	Principal Components	Main Indications	Contraindications	Major Side Effects	Regulatory Status	Refs.
Tisseel	Baxter International Inc.	Human fibrinogen Thrombin solution	Hemostasis, tissue sealing, mesh fixation in hernia repair, alternative or adjunct to sutures or staples	Avoid intravascular administration; caution with spray application	Hypersensitivity/anaphylactic reactions, sensory disturbance, thromboembolic events, air/gas embolism	FDA and EMA approved	[68,69,70,71]
ARTISS	Baxter International Inc.	Human thrombin, human fibrinogen, and an antifibrinolytic inhibitor	Tissue adhesion, skin graft, reconstructive surgery, burns	Avoid intravascular administration; caution with spray application	Hypersensitivity, air or gas embolism, skin graft failure, hematoma	FDA and EMA approved	[71,72,73,74]
VERASEAL™	Johnson & Johnson	Human fibrinogen and human thrombin	Surgical hemostasis, vascular surgery	Avoid intravascular administration; do not use for the treatment of severe or brisk arterial bleeding	Hypersensitivity, allergic reactions, bronchospasm, hypotension, nausea, procedural pain,	FDA approved	[71,75,76,77,78,79]
Beriplast P	CSL Behring GmbH	Fibrinogen Aprotinin solution Human thrombin Calcium chloride solution	Hemostasis, tissue adhesion, suture	Avoid intravascular administration Avoid using it for arterial and strong venous bleeding	Thromboembolic events, anaphylaxis, angioedema, and bronchospasm	EMA approved	[71,80,81]
TachoSil (R)	Corza Medical	Topical fibrin sealant patch consisting of human fibrinogen and human thrombin coated onto an equine collagen sponge	Adjunctive hemostasis and tissue sealing in cardiovascular and hepatic surgery	Avoid intravascular administration	Hypersensitivity reactions, thrombosis, infection, and adhesions	FDA approved	[82,83,84]
Vivostat^®^ Fibrin	Vivostat	Autologous fibrin sealant based on thrombin and fibrinogen	Tissue sealing in personalized surgery	Not reported	Not reported	CE-marked medical device	[85]

### 3.2. Fibrin Hydrogels

Hydrogels are widely used as scaffolds in tissue engineering due to their resemblance to native biological tissues and their key characteristics, including high water content, permeability, and viscoelastic behavior [86,87]. Moreover, hydrogels must be biocompatible and biodegradable, support cell migration, proliferation, and differentiation, provide temporary mechanical support for new tissue formation, maintain a proper microenvironment, and absorb tissue exudates [88,89]. In this context, fibrin-based hydrogels are promising materials for hydrogel manufacturing because they are a major component of blood coagulation and the wound-healing process [86]. Fibrin-based hydrogels are biocompatible, minimally immunogenic, and present non-toxic degradation, especially when derived from autologous sources [90]. Fibrin hydrogels mimic the ECM, providing a structure that supports inherent cell binding and creates appropriate porosity, thereby enabling efficient cell adhesion, spreading, migration, and proliferation [86,89]. Additionally, fibrin-based hydrogels can be used for GF delivery and therapeutic molecules, supporting angiogenesis and tissue regeneration [89]. Their high water content (approximately 95–98%) allows efficient nutrient and waste exchange, further enhancing their suitability as cellular microenvironments [90].

Fibrin-based hydrogels are remarkable due to their tunability. As with the FS, adjusting parameters such as fibrin concentration, thrombin level, ionic strength, pH, and temperature can precisely control the hydrogel’s structural features (e.g., fiber diameter, porosity, and stiffness) [86,88]. Thus, fibrin-based hydrogels can be used in various medical applications, including cell encapsulation, drug delivery systems, and injectable systems [86,89,90]. Additionally, their viscoelastic properties and strain-stiffening behavior mimic the native ECM. These features are important because they can enable hydrogels to maintain mechanical forces while keeping their structural integrity [89,91]. However, these hydrogels are highly biodegradable through two main mechanisms: fibrinolysis (mediated by plasmin) and matrix remodeling (mediated by matrix metalloproteinases (MMP)), which can decrease the efficiency of the hydrogel and decrease the success of engraftment in tissue engineering [86,90]. MMPs, particularly MMP-2 and MMP-9, are present in the chronic wounds environment (e.g., diabetic foot ulcers), contributing to excessive ECM degradation, persistent inflammation, impaired angiogenesis, and delayed healing [92,93]. However, even if MMPs and plasmin play important roles in normal wound repair and facilitate matrix remodeling, their dysregulated activity in chronic wounds accelerates fibrin and ECM degradation. In this context, their excessive synthesis can lead to premature breakdown of fibrin-based biomaterials before tissue regeneration is achieved [94,95]. Thus, these observations highlight the challenges in maintaining the integrity of fibrin-based hydrogels and the need for stabilization strategies.

To reduce fibrinolysis, inhibitors that block the active site of plasmin (e.g., tranexamic acid or epsilon-aminocaproic acid) and aprotinin can be used to inhibit MMP, thereby preventing fibrin breakdown. By modulating degradation rates with plasmin and MMP inhibitors, one can enhance synchronization between scaffold degradation and tissue regeneration [86,90]. However, their weak mechanical and chemical properties often necessitate reinforcement or modification to meet specific clinical requirements [86,89]. For wound-healing applications, hydrogels must be immunologically neutral and exhibit balanced mechanical and chemical properties [10]. Thus, by modifying the chemical and mechanical properties, it is possible to improve and control the polymerization and degradation processes of fibrin-based hydrogels. In addition, biological networks can be mimicked, yielding a fibrin matrix that is key to forming complex 3D structures [90]. To balance mechanical performance and biodegradability, fibrin-based hydrogels can be combined with synthetic or natural polymers to reinforce fibers [86,96]. Natural polymers are preferred for their high biocompatibility and bioactivity, as they are recognized by the human body as macromolecules, such as polysaccharides and proteins, whereas synthetic polymers can considerably improve mechanical properties and biodegradability [10]. Moreover, cross-linking is used to form irreversible covalent bonds, thereby increasing resistance to degradation, whereas physical cross-linking maintains biocompatibility by forming reversible interactions [96,97]. However, despite their advantages, the clinical translation of fibrin-based hydrogels remains limited by rapid degradation and insufficient mechanical strength [98].

Fibrin can form straight, individual fibers that create a denser, more entangled network. However, the denser, less porous structure of the fibrin-based hydrogel can offer hydraulic resistance. Collagen has a much softer and more elastic shear modulus than fibrin. Fibrin stiffness can be attributed to crosslinking by factor XIII, which stabilizes fibrin networks [99]. Additionally, collagen is also a major component of ECM, and collagen hydrogels are commonly used to manage chronic skin wounds due to their controlled drug release and notable properties such as biocompatibility, solubility, high water retention capacity, and sensitivity to pH changes in the surrounding environment [100,101,102]. Its mechanical flexibility and numerous cell-binding motifs enable collagen to promote cellular adhesion, proliferation, migration, and differentiation. However, pure collagen is too weak to sustain physiological loading conditions and can degrade before sufficient tissue regeneration has occurred [102]. The formation of collagen hydrogels requires only the neutralization of an acidic solution, whereas fibrin requires an additional step of enzymatic activation by thrombin. However, the major difference between the two materials lies in their in vivo degradation. Fibrin is a transient material, intended to form and degrade rapidly as part of the healing process. Consequently, the degradation rate of fibrin hydrogels is usually faster than that of collagen (e.g., within a few days if they are not further cross-linked, whereas collagen hydrogels can persist for weeks or even months), strongly depending on formulation, crosslinking strategy, and biological environment [103]. In addition, it was reported that the introduction of collagen into fibrin-based hydrogels can increase matrix rigidity, increase network density, and decrease the hydrogel’s swelling capacity, having the potential to provide structural stability [104].

Alginate is another natural polymer used in the development of fibrin-based hydrogels, but it must be modified to support cellular adhesion. However, alginate is a biomaterial with increased biocompatibility, biodegradability, non-toxicity, non-immunogenicity, easy gelation, and easy availability [105,106]. After cross-linking with calcium ions (Ca^2+^), alginate hydrogels can achieve mechanical properties suitable for certain tissue engineering applications and three-dimensional cell cultures [106]. However, to more accurately replicate extracellular matrix characteristics, alginate is frequently combined with natural biopolymers such as fibrinogen [107]. This combination is facilitated by the shared role of calcium ions, which drive ionic cross-linking of alginate and contribute to fibrinogen gelation, thereby forming a composite hydrogel network with improved structural and biological properties [106,108].

Agarose and hyaluronic acid are frequently used to develop hydrogels due to their biocompatibility and favorable biological properties. Agarose can sustain cell morphology, support cell differentiation (e.g., mesenchymal cells), and exhibit hemostatic properties due to its natural adhesiveness [109,110]. Moreover, its highly hydrated environment helps reduce the inflammatory response; however, the lack of specific cell adhesion sites and its high stiffness limit cell adhesion and proliferation [111].

Hyaluronic acid is a component of the ECM and is known for its low immunogenicity, biodegradability, and extensive potential for chemical modification. In this context, HA is used in fibrin-based hydrogels because it has been reported to stabilize and reinforce the network in these formulations, while fibrin provides essential adhesive signals for cell proliferation and angiogenesis [112]. Although HA does not directly promote cell adhesion, it influences cellular functions by interacting with specific receptors called hyaladherins [113].

In addition, PVA and PEG are widely used due to their biocompatibility, lack of immunogenicity, and adjustable stiffness [114]. Depending on the formulation and fabrication methods, fibrin and PVA can form interpenetrating polymer networks (IPNs) that increase the elastic modulus and promote the formation of stiffer hydrogels, while keeping fibrin on the surface and favoring cell development [90].

Another strategy to improve the properties of fibrin-based hydrogels involves silanizing fibrin networks, yielding nanofibrillar hydrogels with mechanical properties superior to those of unmodified fibrin formulations. These hydrogels are able to preserve the ability to support cell proliferation [115]. Furthermore, the use of silanes with hydrophobic groups can influence gel formation kinetics and rheological properties by improving the dispersion of fibrinogen molecules in the system [116].

Because the wound-healing process is complex and coordinated by interactions among multiple biological factors, including cells, tissues, and molecular signals [117], it involves biological mechanisms responsible for cell migration, proliferation, ECM deposition and remodeling, as well as inflammation and angiogenesis [117,118]. Minor wounds can heal rapidly, but extensive injuries take longer to heal, which can lead to fibrotic scar formation or impaired function of the injured tissues [117]. The healing process implies four distinct phases: hemostasis, inflammation, proliferation, and maturation (remodeling) [119]. Any disruption that occurs during the healing process can hinder healing and thus lead to chronic wounds, which are associated with various comorbidities, including diabetes, vascular insufficiency, or inflammatory disorders that promote prolonged inflammation [120,121]. Although inflammation is an essential step in the healing process, increased production of reactive oxygen species (ROS) can damage tissue and hinder healing [120]. In general, the chronic injury-healing process is blocked during the inflammatory and proliferative phases, favoring infections, poor vascularization, and necrotic tissue formation [121]. In burn injuries, wound healing is more complex and is mainly influenced by immune responses. For a burn wound to heal, three stages must occur successively: inflammation, granulation tissue formation, and remodeling [122]. Large or deep burns can trigger widespread inflammation, increasing the risk of infection, sepsis, and multi-organ dysfunction, which requires a prolonged time for healing and increases the mortality rates [10,122]. In addition to injury severity, both local (e.g., infection, oxygenation) and systemic (e.g., age, obesity, ischemia) factors further modulate healing outcomes, either supporting or hindering tissue repair [122,123]. Given the challenges regarding chronic wounds and burn injuries therapies, fibrin-based composites started to gain attention as possible therapeutic approaches. Due to fibrin’s implication in biological processes in hemostasis and tissue repair, fibrin-based systems can be engineered to offer promising strategies to improve healing [35].

**Figure 2:**
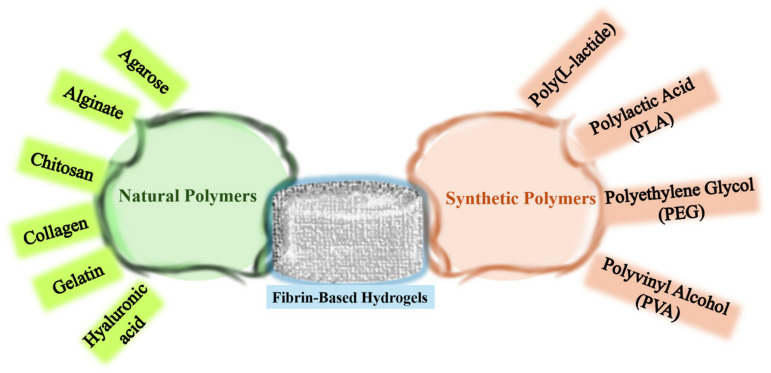
Natural and synthetic polymers used in the fabrication of fibrin-based hydrogels. Created based on the information from [109,110,114,124].

Table 2 summarizes various types of fibrin-based hydrogels used in wound healing. This table highlights the composition of the materials used to fabricate hydrogels designed for wound healing, along with their properties and potential biological activity. Additionally, the materials analysis suggests a need to improve the mechanical properties of fibrin-based hydrogels. Even if bare fibrin hydrogels generally provide an optimal environment for cell migration and proliferation, their performance depends on fibrin composition, network architecture, and experimental conditions. In addition, these materials exhibit limited mechanical properties, including low strength and stress-induced collapse. To overcome these limitations, researchers focused on using various adjuvant materials exemplified in Figure 2. Therefore, the table highlights a balance between improving material performance and maintaining biocompatibility.

**Table 2:** Fibrin-based hydrogels for wound healing applications.

Fibrin-Based Material	Key Components	Main Properties	Biological Activity	Limitations	Applications	Refs.
Fibrin hydrogel	Fibrinogen, thrombin, FXIII, Ca^2+^	Creates a dense network with low permeability. It can retain pressure.	Supposed to support cell migration, adhesion, and nutrient transport, and ECM-like behavior	Has weak mechanical properties that influence the cell environment Collapses under stress	Wound healing 3D models	[99]
Fibrin + silica/chitosan-silica NPs	Fibrin + Silica NPs Fibrin + Silica-Chitosan NPs	Improved mechanical properties. NPs reinforced the fibrin network.	Improvement in fibroblast growth in fibrin hydrogels and silica-chitosan NPs compared to fibrin hydrogels and silica NPs	Dose-dependent toxicity on fibroblasts due to the presence of silica NPs.	Wound dressings, tissue engineering	[125]
Alginate–fibrinogen- based hydrogel + nisin	Alginate, fibrin, nisin, EDTA	Stable 3D hydrogel network, 2200% swelling capacity was demonstrated Improved moisture retention Improved barrier function	Antimicrobial effect (strong) due to nisin encapsulation and its sustained release Improved healing Collagen formation ~97% wound closure was demonstrated in a rat full-thickness skin wound model, exceeding both the alginate-only and gauze control groups	Limited mechanical strength, Need for chemical modification	Wound healing	[126]
Chitosan/fibrin + silver NPs	Chitosan, fibrin, AgNPs, mupirocin	Antibacterial effect Sustained release of mupirocin Bilayer structure Improved mechanical strength Porous structure	Strong antimicrobial effect Reduced inflammation Improved healing near-complete re-epithelialization (97%) in Albino Wistar rats compared to the control (62%) and chitosan-fibrin (86%)	Dose-dependent toxicity due to the presence of silver NPs	Drug delivery system Infected wound healing	[127]
Fibrin + SMPDA NPs	Fibrin + SMPDA NPs	Stimuli-responsive hydrogel Nitric oxide-releasing NPs, Improved antimicrobial properties Swelling behavior, 3.5 swelling ratio Completely degraded after approximately 7 days Excellent injectability Rapid gel formation	Antimicrobial and anti-inflammatory properties Enhanced healing in *S. aureus*-infected full-thickness skin wounds (90%) by day 12, compared to ~53–80% closure in control groups.	Nitric oxide release relies on external stimulation, compromising its applicability in certain clinical scenarios	Infected wounds	[128]

Furthermore, given the findings presented in Table 2, Table 3 aims to provide a more comprehensive analysis of advanced fibrin-based materials for other biomedical applications, such as tissue engineering and regenerative medicine. Research has focused on using various polymers to introduce structural modifications that overcome the intrinsic limitations of fibrin, thereby extending its functionality beyond wound repair to encompass more complex tissue regeneration strategies. In this respect, natural polymers such as agarose, hyaluronic acid (HA), collagen, alginate, and chitosan, as well as synthetic polymers such as polyvinyl alcohol (PVA) and polyethylene glycol (PEG), are combined with fibrin to create composite materials with mechanical and structural properties tailored to various applications. It should be noted that some of the included research studies focus on design and biomaterial development, while others include in vitro and in vivo biological evaluation. However, the studies differ in their objectives, experimental designs, and stages of development, and some require further investigation of specific quantitative, biological, or clinical parameters.

### 3.3. Platelet-Derived Fibrin Matrices

#### 3.3.1. Platelet-Rich Plasma-Derived Fibrin

Platelet-Rich Plasma (PRP) represents the first generation of autologous blood-derived product enriched with high concentrations of platelets, GF, and bioactive proteins, promoting tissue repair, regeneration, and immunomodulation [137,138,139,140]. It is safe to use in clinical practice due to its ease of preparation and versatility. PRP can be delivered as an injectable formulation to the wound site or as a solid fibrin matrix that forms after activation, allowing it to function both as a drug delivery system for biomolecules and as a structural scaffold that supports tissue repair [137,141].

The therapeutic effect of PRP is attributed to its high content of GF and signaling proteins. In this context, PRP promotes key biological processes, including cell proliferation, migration, angiogenesis, and ECM formation. Moreover, PRPs are known for their ability to slow fibrin degradation, thereby favoring a sustained release of bioactive molecules and resulting in improved tissue repair [137]. The use of PRP as a fibrin-based scaffold is achieved by activating it with agents such as calcium chloride, thrombin, or collagen, forming an ECM that mimics the natural one. However, their performance is dependent on the formulation and preparation method. The platelet concentration, fibrinogen, leukocyte content, and the type of activators used [139,142]. In PRP-derived fibrin matrices, limitations such as relatively slow clot formation, weak mechanical performance, poor tissue adhesion, and the tendency of the fibrin clot to retract are the reasons for PRP’s reduced structural stability that results in premature release of GF [140,143]. A strategy to address these limitations involves modifying PRP’s composition. Chitosan and hyaluronic acid have been shown to improve scaffold integrity, prolong GF release, and enhance regenerative outcomes. These strategies aim to optimize both the biological and mechanical performance of PRP-based systems for specific clinical applications [137,139].

To obtain PRP, anticoagulated blood is centrifuged to separate the components into plasma, buffy coat, and red blood cell layers, and then platelets are concentrated in the plasma fraction. However, the preparation method can yield different compositions, which can affect therapeutic effectiveness, underscoring the importance of standardization [144].

Despite this, PRP remains one of the most widely used materials in regenerative medicine, due to its biological activity and adaptability. As for their limitations, research is ongoing to develop improved formulations and alternative platelet-based biomaterials to enhance clinical outcomes [139]. In this context, platelet-rich fibrin was introduced as a second-generation platelet concentrate that aims to eliminate the need for anticoagulants during manufacturing. Platelet-rich fibrin forms the clot after centrifugation and is capable of a slower GF release over time, favoring a natural healing process [140].

#### 3.3.2. Platelet-Rich Fibrin Matrices

The second generation of platelet concentrates is platelet-rich fibrin (PRF), which is widely used in regenerative medicine for its simplicity, autologous nature, and favorable biological activity [145,146,147,148]. As with PRP, PRF is obtained by centrifugation without anticoagulants, allowing the natural formation of a clot [149,150]. This results in a fibrin matrix enriched with platelets, leukocytes, cytokines, and circulating stem cells, mimicking the physiological environment of wounds and facilitating healing [148]. Thanks to this property, the need for biochemical manipulation is avoided, making PRF a cost-effective and clinically accessible material [150,151].

PRF is structurally composed of a dense fibrin matrix containing cellular and molecular components such as platelets, white blood cells, GF, immune cytokines, and circulating stem cells [145,152]. PRF might form a more flexible and organized structure than PRP, and, due to its slower fibrin clot formation, the GF are efficiently trapped within the fibrin matrix and released over time, supporting long-term healing [145]. In this type of structure, platelets play an essential role as biological mediators by releasing a wide range of GF (e.g., PDGF, VEGF, TGF-β, EGF, and IGF-1) and cytokines (e.g., IL-1β and IL-4) [53,146,153]. PRF has been associated with multiple therapeutic effects that significantly improve healing outcomes, including anti-inflammatory properties, the capacity to promote the transition of macrophages from a proinflammatory (M1) to a regenerative (M2) phenotype, and antimicrobial effects conferred by platelets and leukocytes. PRF is associated with reduced postoperative pain, improved hemostasis, and faster tissue regeneration [153]. However, the reproducibility of these effects depends on centrifugation conditions, cell composition, fibrin architecture, and preparation protocols, and results can vary from one study to another [154,155,156,157,158].

PRF has been shown to promote wound healing and to act as a structural scaffold with bioactive signaling properties, capable of generating a fibrin network. Thus, PRF induces matrix formation and reduces inflammation, which can be an advantage for chronic wound healing [146,159]. Additionally, their performance can be enhanced by integrating biomaterials, such as polymers, to create materials for tissue engineering [146]. PRF-based hydrogels, used in wound healing, are presented in Table 4. The main applications of such materials are in chronic wounds, where they are characterized by their ability to release GF in a sustained manner. This property stimulates angiogenesis, cell proliferation, and tissue remodeling, improving healing outcomes for these types of wounds. The results showed improved and complete re-epithelialization and reduced inflammation. However, limitations include a lack of long-term studies, small sample sizes, and potential compatibility issues. In addition, direct comparisons between studies remain challenging because PRF formulations depend on centrifugation parameters, preparation protocols, cellular composition, and fibrin architecture. Variations in these factors may significantly influence their biological properties and therapeutic performance.

As with PRP, PRF is limited by variability in the preparation protocol. In this regard, parameters such as centrifugation speed and duration can considerably affect the structure, density, and, implicitly, biological activity. Therefore, standardization of preparation methods remains an important challenge for ensuring reproducibility and optimizing clinical performance [150].

**Figure 3:**
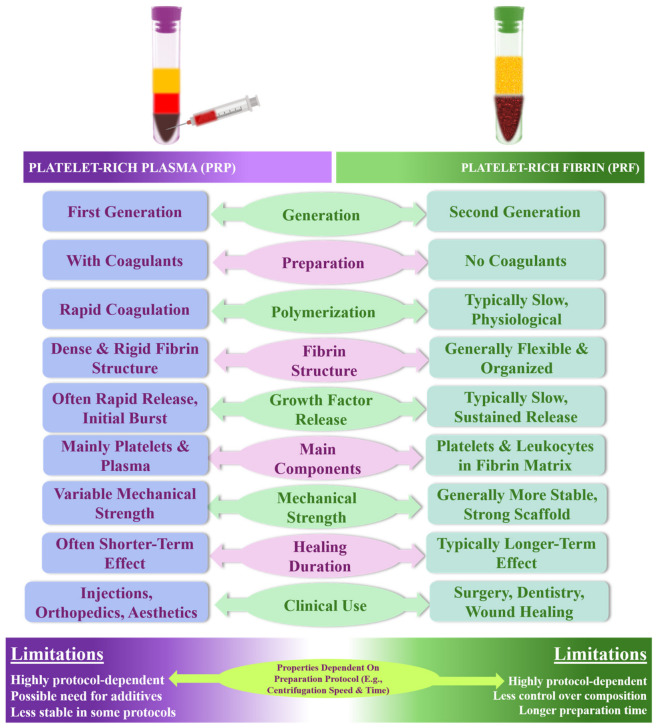
Schematic comparison between platelet-rich plasma (PRP) and platelet-rich fibrin (PRF) and their key differences in structure, release profile, and clinical use reported in the literature. Created based on the information from [140,163,164].

Figure 3 highlights the main differences between PRP and PRF regarding their structural features, biological activity, and clinical performance. Both are autologous platelet concentrates designed for tissue engineering applications, but their preparation methods yield distinct therapeutic profiles. PRP is prepared using anticoagulants and other activators, leading to rapid fibrin polymerization. Thus, PRP forms a dense, rigid network with rapid GF release, whereas PRF forms a flexible, organized matrix that maintains sustained, controlled GF release, supporting longer healing and tissue regeneration. PRP is mainly used for injections, while PRF also acts as a scaffold that promotes cell growth and offers anti-inflammatory and antimicrobial benefits. The illustrated differences represent typical characteristics and may vary depending on preparation protocols, centrifugation parameters, cellular composition, and fibrin architecture.

#### 3.3.3. Clinical Applications of Fibrin-Based Materials

The development and clinical implementation of fibrin-based materials have evolved, and they were approved by the Food and Drug Administration (FDA) in 1998 as a sealant formulation, being capable of functioning simultaneously as a hemostat, sealant, and adhesive [165]. In this regard, Table 5 summarizes registered clinical trials identified on ClinicalTrials.gov, reflecting the growing interest in fibrin-based materials in the management of chronic wounds and burn-related injuries. The majority of the presented studies are interventional and are in the incipient or intermediate phases, and the therapeutic approaches include combinations of fibrin with stem cells, platelet-rich plasma, or other advanced therapies. Moreover, the table includes the study objectives, planned endpoints, development phases, and the recruitment status. However, the clinical evidence remains limited, with many studies still ongoing, recruiting,, completed without published results, or not yet reporting any outcomes.

Beyond the application of fibrin-based materials in wound healing and soft tissue regeneration, these materials can also be used in other applications, including regenerative medicine, plastic surgery, and neurosurgery [166,167]. Moreover, fibrin-based materials have been extensively investigated for cartilage and bone regeneration. In this context, Table 6 presents a selection of clinical studies available on ClinicalTrials.gov that investigate the application of fibrin-based materials in cartilage and bone regeneration. The researcher’s interest in developing advanced regenerative strategies, particularly for knee injuries and joint cartilage, has increased. The advancement from interventional phases to Phase 3 clinical evaluation assesses their clinical potential. Furthermore, the use of fibrin as a biological scaffold or adhesive is a common theme, highlighting its essential role in facilitating tissue regeneration.

Nevertheless, interpretation of these findings should be approached with caution, as several studies were terminated, and many have not yet reported results. Therefore, additional well-designed clinical studies are required before definitive conclusions can be drawn regarding efficacy and safety. These applications were included because they can demonstrate the broader regenerative potential of fibrin-based materials.

Table 7 highlights other clinical applications involving fibrin-based materials in regenerative medicine, including the use of PRF for postoperative healing and fibrin adhesives in bone and neurological reconstructions. Studies indicate that fibrin-based materials are valued for their enhanced biocompatibility and capacity to support tissue regeneration, resulting in favorable clinical outcomes. These results highlighted the material potential to reduce the complications associated with the intervention and successful interventions. However, these studies remain limited and individual, underscoring the need for further large-scale studies.

## 4. Advantages, Limitations, Safety Considerations, and Future Perspectives

Fibrin-based materials are preferred in applications such as wound healing and soft tissue regeneration due to their key advantages, including increased biocompatibility, biodegradability, and resemblance to the native ECM [86,89]. Moreover, these materials have been widely reported to support cell adhesion, migration, proliferation, and differentiation, and stimulate angiogenesis and tissue remodeling. Depending on their formulation and the incorporation of bioactive compounds, fibrin-based hydrogels can achieve new functionalities, including carriers for cells, drugs, and GF [86]. Furthermore, their rapid in situ gelation also makes them attractive for minimally invasive regenerative therapies [90].

**Figure 4:**
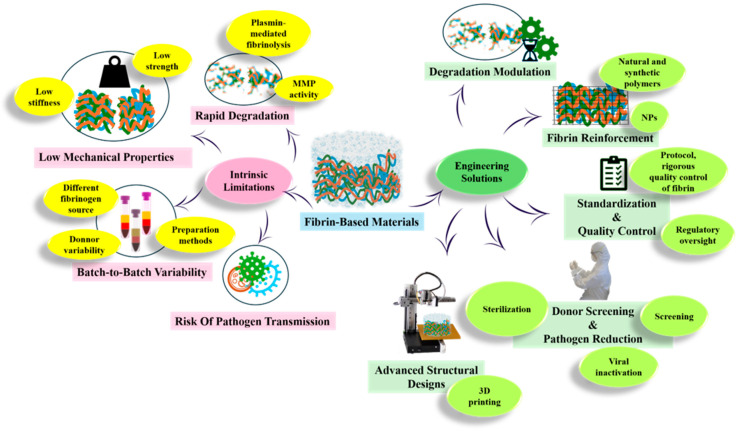
Overview of limitations of fibrin-based materials and the strategies proposed to overcome them.

However, fibrin-based materials also present several limitations (Figure 4). In this context, unmodified fibrin-based materials exhibited poor mechanical strength, low long-term stability, shrinkage during gel formation, and rapid degradation due to fibrinolysis or matrix metalloproteinase activity [86,96]. Additionally, materials derived from fibrin products can exhibit batch-to-batch variability, thereby compromising reproducibility. Studies have shown that these variations can result from changes in preparation methods or fibrinogen sourcing [86]. These drawbacks may restrict their application in load-bearing tissues or in situations requiring prolonged structural support [89,90].

Safety concerns are associated with a specific class of fibrin-based materials. In particular, a limitation of plasma-derived products is that they pose a residual risk of pathogen transmission and elicit immunological reactions in patients, including pyrogenesis, during bioresorption [171,172]. Safety considerations vary depending on the source of fibrin and the manufacturing process. Autologous preparations are generally considered safer with respect to infectious disease transmission, whereas homologous plasma-derived products rely on extensive screening and purification procedures to ensure safety [173]. This limitation can be mitigated by rigorous screening of blood donors and plasma pairs, as well as related sterilization steps [96]. Even though these measures can improve the safety of fibrin-based materials, the risk of pathogen transmission still exists. Thus, the lack of sensitivity and specificity in current screening methods, the lack of specific tests for certain types of pathogens, and the fact that many of these pathogens are not routinely tested for make the use of fibrin-based materials challenging, since low concentrations of pathogens do not necessarily imply a low risk to the patient [174]. In clinical practice, the use of fibrin-based materials as sealants is limited by the application method (spray or barrel syringe), which can alter their properties. Moreover, thrombin concentration is very important in such cases. Even though a high thrombin concentration can accelerate polymerization, it can also produce extremely dense networks that inhibit cell infiltration and migration. In this context, research should focus on developing fibrin-based materials that enable both permissive cell infiltration and regeneration while exhibiting mechanical properties appropriate to the specific application [95].

Furthermore, as shown in Table 1, most commercially available FSs have limitations, including adverse effects associated with their use, such as thrombosis after intravascular administration, hypersensitivity reactions, and gas embolism during spray application. For example, some commercially available FSs contain synthetic aprotinin, which can cause hypersensitivity or anaphylactic reactions, especially after repeated exposure. Moreover, FSs can be associated with graft loss due to excessive application and with product degradation by certain chemicals [172]. Moreover, fibrin is well tolerated, but any modification can alter its immunogenicity, potentially due to the introduction of chemical reagents, the generation of new functional groups, and a low degradation rate [90,96].

However, depending on the source of fibrin, fibrin-based products still require donor screening, quality control procedures, manufacturing standardization, and regulatory oversight to ensure product safety, consistency, and reproducibility.

**Figure 5:**
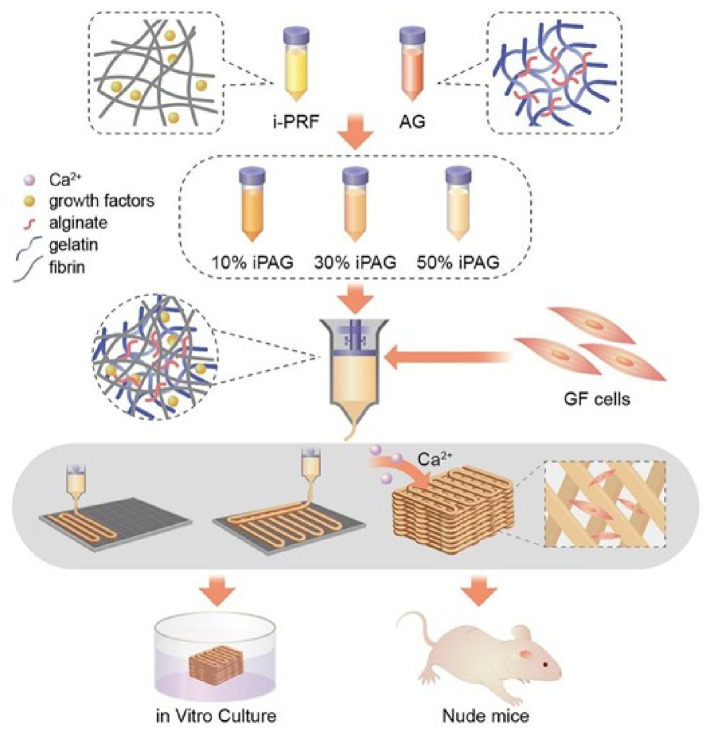
Schematic representation of the obtaining process of the 3D bioprinted construct performed by Yi et al. Reprinted from an open-access source [178].

Future perspectives on fibrin-based materials focus on overcoming limitations through advanced strategies. This review has already discussed some approaches to improve the properties of fibrin-based materials. Chemical and physical cross-linking improve the structural stability of the materials. The use of synthetic and natural polymers, nanoparticles, and 3D-printed scaffolds can enhance mechanical strength and regulate degradation kinetics. In this context, 3D bioprinting represents an approach that can create 3D shapes and multiplex constructs that mimic the structural features of tissues [175]. However, it should be noted that the homogeneity of bioinks is a key factor affecting the printing process and the quality of the final product [176]. Thus, the bioinks should have a shear-thinning behavior. The bioink’s viscosity should decrease under the shear forces acting during extrusion and also quickly regain its consistency after deposition, thereby ensuring that the printed structure retains its shape [176,177]. Moreover, the mechanical and rheological properties of the bioink are crucial for maintaining the construct’s structural integrity after printing. In this regard, bioinks should exhibit rheological, mechanical, biological, and functional properties appropriate for the desired tissue application [97]. Depending on the formulation and testing conditions, fibrin-based bioinks generally exhibit predominantly elastic behavior rather than viscous behavior, so they need to be combined with other polymers to improve the structural stability of bioprinted structures. These limitations can be overcome by adjusting the concentrations of fibrinogen, thrombin, and other components to control the bioink’s viscoelasticity. In addition, the gelation mechanisms can also be adjusted to achieve immediate stabilization of the structures after printing. In this way, precise, functional three-dimensional constructs can be produced to support effective tissue regeneration [177]. For example, Yi et al. [178] investigated the regeneration of oral soft tissue using a 3D-bioprinted material (Figure 5) based on injectable PRF composed of i-PRF, alginate, and gelatin. The combination of these natural polymers in the resulting bioink improves structural stability and handling properties, maintains shape well, and exhibits a porous structure favorable for tissue engineering. Moreover, incorporating the PRF 3D-printed structure resulted in prolonged, sustained GF release.

In the same manner, Cavallo et al. [176] developed a bioink composed of fibrinogen, alginate, and calcium chloride for skin equivalents intended for wound-healing and regenerative-medicine applications. The bioink demonstrated excellent printability, shape fidelity, and mechanical stability, maintaining defined geometries and properties suitable for tissue applications. Both of these studies on fibrin-based bioinks are promising platforms for tissue engineering applications that sustain GF delivery, reduce inflammation, enhance angiogenesis, and improve tissue regeneration.

Another challenge for bioinks destined for clinical applications is sterilization, as it must ensure the removal of contaminants while maintaining cell viability and material properties [176]. Conventional methods, such as autoclaving or gamma- and electron-beam irradiation, can cause denaturation and degradation of protein scaffolds, thereby affecting their mechanical and functional properties. Therefore, choosing an appropriate sterilization method is essential for preserving the performance of bioinks [176,179].

However, future research is still needed to enhance the biological complexity, reproducibility, and clinical relevance of fibrin-based materials. Researchers need to focus on long-term mechanical stability, degradation rate, and future in vivo tests to assess safety, material integration, vascularization, immune response, and the material’s applicability in further clinical trials. In addition, the clinical translation of fibrin-based bioinks is constrained by regulatory requirements related to manufacturing standardization, quality control, sterility assurance, and long-term safety evaluation. Ultimately, combining bioinks and fibrin-based materials with patient-derived cells and personalized 3D bioprinting strategies may enable the development of customized approaches for applications such as tissue engineering, skin substitutes, chronic wound treatments, and reconstructive surgery.

## 5. Conclusions

Given the urgent need to address chronic wound healing and soft tissue defects, which are associated with major complications, fibrin-based materials are considered a promising approach due to their combined natural biological functionality and versatile engineering potential. Their remarkable properties, including their ability to support hemostasis, provide a temporary ECM, regulate cellular responses, and deliver therapeutic agents, make them particularly valuable for complex and chronic wounds. For this reason, fibrin-based materials, particularly fibrin sealants and certain platelet-derived products, have achieved clinical translation and are already being used in medical applications. In contrast, some of the presented studies are still in the preclinical phase or on the design, synthesis, and characterization of physicochemical properties. Furthermore, clinical studies and clinical trials indicate a growing interest in the potential of fibrin-based materials for medical applications. However, clinical evidence remains limited, so further well-designed studies are required to confirm their efficacy and long-term safety. Yet, some limitations must be overcome for these materials to be used on a large scale. Future progress will likely depend on integrating fibrin with natural or synthetic polymers, nanoparticles, stem cells, and additive manufacturing technologies such as 3D bioprinting. In this regard, fibrin-based materials hold promise for personalized regenerative medicine, having the potential to offer safer and more effective solutions for wound management and tissue reconstruction.

## Figures and Tables

**Table 3:** Fibrin-based hydrogels in advanced biomedical applications.

Fibrin-Based Material	Composition and Design Strategy	Structural and Mechanical Features	Biological Functionality	Cellular Response	Limitations	Target Application	Refs.
Fibrin-agarose skin substitute	Fibrin + agarose + fibroblasts + keratinocytes	Biomimetic structure Layered structure	Supports angiogenesis Improve dermal regeneration Promotes membrane formation and integration with the host tissue	ECM remodeling, skin regeneration	Rapid tendency of fibrin (alone) degradation Need to be combined with agarose	Skin substitutes	[129]
PEG-Fibrin Hybrid Hydrogel	PEG + Fibrin	Tunable mechanical properties (e.g., stiffness) Tunable porous structure Fibrin was not completely released from the hydrogel matrix Mechanical stiffness increased with fibrin concentration	Bioactive Mechanically stable due to PEG scaffold	Improve tissue formation Enable healing cascade	Need for a synthetic and bioinert polymer to improve the material properties	Vascularized tissue engineering	[130]
Fibrin-PVA scaffold	Fibrin + PVA	Porous structure with interconnected pores (~330 μm) Improved mechanical properties (e.g., tensile strength) Tunable degradation rate PVA significantly improves resistance to enzymatic degradation (~5 days)	Improved vascularization Promote collagen deposition Enhanced re-epithelialization Wound closure 97% in a full-thickness mouse wound compared to 92% in controls	Enable cellular adhesion, proliferation, and infiltration in the 3D scaffold (mesenchymal stem cells)	Crosslinkers increase the risk of toxicity	Skin tissue engineering	[131]
Collagen-HA-Fibrin Hydrogel (CHAF)	Collagen + HA+ Fibrin + PEG crosslinker	Interconnected pores Open architecture Pore range: 17–435 μm 1182 ± 202% water uptake The hydrogel gradually degrades while maintaining structural integrity	Excellent fibroblast viability and proliferation Supports melanoma spheroid culture	Demonstrated high viability Enhanced proliferation, Normal morphology	Low stiffness	Tissue engineering, Tumor models	[132]
Fibrin-Hyaluronic Acid Scaffold	Fibrin + HA	Dense network Improved stiffness Slow degradation rate	Improved ECM signaling and stability	Improved proliferation, attachment, and spreading Fibrin significantly improves biological remodeling and vascularization	Still needs crosslinking optimization	Skin tissue engineering, Drug delivery	[133]
Alginate Particle-Fibrin Hydrogel	Alginate particles + Fibrin	Porous and fibrous structure Stability Injectable system	Sustain the formation of new tissue	Promotes adhesion, migration, proliferation of fibroblasts, and angiogenesis Formation of new soft tissue in vivo in athymic mice models	Fabrication process requires optimization Low mechanical strength	Soft tissue reconstruction	[134]
Chitosan–Fibrin-Nanocurcumin Hydrogel	Chitosan + Fibrin + Nanocurcumin	Injectable system, Promote controlled release The release profile followed: Fickian diffusion	Stimulates NO production and angiogenesis	Good endothelial cell growth and viability	Reduced cell viability at prolonged culture times due to potential overconfluence	Vascular tissue engineering, Drug delivery	[135]
MSC Spheroid–Fibrin System	Fibrin hydrogel-tuned MSC spheroids	Tunable mechanical properties (stiffness) Higher fibrinogen and optimized salt concentrations produced much stiffer hydrogels	Controls VEGF and PGE2	Enhanced vascularization, macrophage modulation	Mechanism not fully understood	Advanced regenerative therapy	[136]

**Table 4:** Platelet-enriched fibrin hydrogels for wound healing applications.

Fibrin-Based Material	Key Components	Main Properties	Biological Activity	Limitations	Applications	Refs.
i-PRF hydrogel	Platelet-Rich Fibrin + Sericin-Based Hydrogel	Injectable hydrogel Offers sustained GF release	Promote angiogenesis and M2 macrophage polarization	Limited long-term studies	Diabetic wound healing	[160]
Platelet Lysate (PL)-loaded fibrin scaffold	PL + Fibrin	Improved mechanical properties Prevent fibrin from collapsing Promote GF release	Improve faster healing compared to scaffolds without PL and standard wound dressing Promote complete re-epithelialization Promotes collagen formation Anti-inflammatory effect	70% complete healing	Chronic wounds	[161]
Plasminogen-loaded fibrin scaffold	Fibrin + Plasminogen	Designed for controlled, sustained release Nanofibrillar structure	93% wound closure Complete re-epithelialization Improved collagen deposition Reduced inflammation	Small sample size Need for larger human studies	Drug delivery systems Wound healing	[162]
PRP + oxidized alginate hydrogel	PRP, Fibrin, Alginate	Injectable hydrogel Promote dual-phase GF release Improved mechanical and physicochemical properties	Biocompatibility Improved cell proliferation and adhesion Supports ongoing repair and improved wound healing	Xenogeneic mismatch issues	Diabetic wounds	[143]

**Table 5:** Registered clinical trials investigating fibrin-based materials in chronic wound therapy available on ClinicalTrials.gov as of 3 April 2026.

ClinicalTrials.gov ID	Conditions	Intervention/ Treatment	Trial Objective	Study Type	Phase	Recruitment Status	Endpoints	Published Results Available
NCT06843122	Diabetic foot ulcer	Allogenic ADSC cells in fibrin solution	Investigation of the efficacy and safety of allogenic ADSC cells in fibrin solution	Interventional	Phase 2	Not recruiting	Wound size after the first administration Type, frequency, and severity of adverse effects Early efficacy Long-term efficacy and safety Wound healing dynamics	Not available
NCT06810726	Diabetic foot ulcer	Autologous PRF	Investigation of the efficacy, safety, and clinical performance of Autologous PRF	Interventional	Phase 2	Recruting	Complete wound closure, wound size reduction, time of wound closure, and safety	Not available
NCT06319287	Diabetic foot ulcer	PEP/TISSEEL (fibrin sealant)	Evaluation of the safety and efficacy of PEP/TISSEEL (Fibrin sealant) compared to the standard of care	Interventional	Phase 2	Completed	Wound closure, safety, size reduction of wounds at 12 weeks, and pain over 12 weeks	Results posted on ClinicalTrials.gov
NCT05979584	Diabetic foot ulcer	Platelet fibrin Plasma vs. PRP	Comparison of the efficacy and safety of Platelet Fibrin Plasma vs. PRP	Interventional	Not Applicable	Completed	4-week wound area reduction 8-week wound healing rate Total blood volume required Adverse effects Infection evaluation	Not available
NCT05850611	Diabetic foot ulcers	Methylene blue and platelet-rich plasma-fibrin glue	Evaluation of MB and PRF on wound healing in patients with nonhealing diabetic foot ulcers	Interventional	Early Phase 1	Unknown	Ulcer healing rate, wound size, blood pressure, and improvement of hypoxia	Not available
NCT05483777	Diabetic foot	PRF vs. standard dressing	Examination of the effect of PRF application on wound healing	Interventional	Not Applicable	Unknown	Wound healing progression and clinical evaluation Healing of diabetic foot wounds	Not available
NCT00852995	Venous ulcer	HP802-247 + fibrin	Investigation of the effectiveness of two dosing frequencies and two different concentrations of HP802-247 + fibrin	Interventional	Phase 2	Completed	Ulcer healing area, ulcer pain, and dose–response efficacy	Results posted on ClinicalTrials.gov
NCT06664268	Hypertrophic scars Burn scar patients	Plasma rich in growth factors (PRGF-Endoret) Fractional CO_2_ laser-assisted PDT	Comparison between PRGF-Endoret and Fractional CO2 laser therapy	Interventional	Early Phase 1	Not recruiting	Improvement in scar thickness, texture, and appearance	Not available
NCT03113747	Second- or third-degree burns	ALLO-ASCs: allogeneic MSCs +collagen- or fibrin-derived hydrogels	The evaluation of the safety and efficacy of ALLO-ASCs: allogeneic MSCs +collagen- or fibrin-derived hydrogels	Interventional	Phase 1 Phase 2	Unknown	Degree of wound healing, epithelization dynamics, duration of treatments,	Not available
NCT00181974	Burns	Tisseel fibrin sealant	The evaluation of the effectiveness of a fibrin glue in burn surgery	Interventional	Not Applicable	Completed	Hemostasis, skin graft fixation, wound healing, cosmetic outcome	Not available
NCT00161759	Burns	Sheet skin grafts affixed with fibrin sealant	The evaluation of the safety and efficacy of fibrin sealant, compared to skin grafts in burns	Interventional	Phase 1 Phase 2	Completed	Not reported	Not available

**Table 6:** Clinical studies registered on the use of fibrin-based materials in the treatment of cartilage and bone injuries, available on ClinicalTrials.gov as of 10 April 2026.

ClinicalTrials.gov ID	Conditions	Intervention/ Treatment	Trial Objective	Study Type	Phase	Recruitment Status	Endpoints	Published Results
NCT04785092	Cartilage Damage Cartilage Disease	Autologous Cartilage Regeneration: Prelevated cartilage + platelet concentrate and autologous fibrin glue	The evaluation of the clinical performance of the AACR technique in cartilage defects	Interventional	Not Applicable	Completed	Changes in knee functionality, quality of cartilage, and MRI evaluation	Not available
NCT04236739	Cartilage Damage	A mixture of allogenic MSC’s and autologous chondrons with a fibrin cell carrier (Tisseel^®^)	To evaluate the clinical improvement of the knee injury and osteoarthritis	Interventional	Phase 3	Completed	Structural changes in the cartilage	Not available
NCT01400607	Articular Cartilage Disorder Articular Cartilage Degeneration Chronic Articular Injury Acute Cartilage Injury Defect of Articular Cartilage	Neocartilage Implant is surgically implanted and affixed to the subchondral bone using commercial fibrin	The evaluation of the safety and efficacy of the Neocartilage Implant	Interventional	Phase 3	Terminated	Knee injury and osteoarthritis outcomes scores	Not available
NCT04543630	Jaw, Edentulous, Partially Diabetes	Advanced platelet-rich fibrin and autogenous bone graft	The objective of the study is to compare the outcomes of implant treatment	Interventional	Not Applicable	Not recruiting	Peri-implant marginal bone level, the stability of the graft	Not available
NCT04537013	Knee Injuries Cartilage Injury Cartilage Disease	Debridement of cartilage followed by placement of small holes in the affected area to fill the debrided area with marrow and cells, followed by affixing Chondro-Gide^®^ with fibrin glue.	The evaluation of the efficacy and safety of an investigational treatment for large chondral lesions of the knee, compared with standard treatment	Interventional	Not Applicable	Terminated	Knee injury and osteoarthritis outcome score, MRI observations	Not available
NCT01532076	Osteoporotic Fractures	Cellularized composite graft augmentation: -liposuction, cell isolation, embedding of SVF cells in fibrin gel, wrapping around hydroxyapatite granules Acellular composite graft augmentation: -Open reduction and internal fixation using acellular augmentation with fibrin-embedded granulated hydroxyapatite	The evaluation of whether the augmentation of bone defects can reduce the complication rate following proximal humerus fractures	Interventional	Phase 2	Terminated	Development of secondary dislocation, functional outcome, safety, bone mineral density, dose–response	Not available
NCT01230931	Fracture Fixation Intra-Articular Fracture	Vitagel (by Stryker)	The investigation into whether the hemostatic agents applied topically during surgery for acetabular fractures can reduce blood loss	Interventional	Not Applicable	Terminated	Intra-operative rate of blood volume loss, hemoglobin levels monitoring, and volume of blood products	Results posted on Clinical-Trials.gov

**Table 7:** Clinical applications of fibrin-based materials in regenerative medicine.

Material	Composition and Design Strategy	Clinical Outcomes	Limitation	Clinical Application	Refs.
Platelet-Rich Fibrin (PRF)—gel/membrane	Autologous fibrin matrix rich in platelets and leukocytes	Reduced fistula rate to 6% after laryngectomy	Limited large-scale studies	Post-surgical healing (laryngectomy)	[168]
Fibrin adhesive for orbital reconstruction	Autologous bone graft + fibrin sealant (adhesive fixation)	Improved enophthalmos, resolved diplopia, no complications	Case study (single patient)	Orbital floor reconstruction	[169]
PRF multilayer system (skull base repair)	Solid PRF membranes + injectable PRF (layered system)	~95% success in preventing CSF leaks, no complications	Needs further validation	Neurosurgery (sellar floor reconstruction)	[170]

## Data Availability

No new data were created or analyzed in this study.
